# M. Tacheron's Pathological Researches

**Published:** 1824-03-01

**Authors:** 


### Medico-Chirurgical Review,

AND

### JOURNAL OF MEDICAL SCIENCE.

( Analytical Series. )

" Nec Aranearum textus ideò melior, quia ex se fila fingunt; nec noster vilior, quia ex alienis libamus, ut apes."

Vol. IV.] MARCH 1, 1824. [No. 16.

### I.

### *Recherches Anatomico-Pathologiques sur la Médecine Pratiques.* Par M. Tacheron.

[Second Analytical Article.—Continued from page 511.]

Sect. I. *Enteritis.*—M. Tacheron defines Enteritis to be " inflammation of the whole or a part of the mucous membrane—or of all the membranes (ou de toute l'épaisseur) of the small or large intestines." According as the phlegmasia predominates in one or other of these structures, or parts, the characteristic symptoms will appear; especially if the disease be acute.

Enteritis may be occasioned by intus-susception—by indurated fæces—by spasm—external pressure—herniæ—poison—by acrid substances taken into the stomach—drastic purgatives—even gentle laxatives, given untimely—by metastases, or translations of external inflammations—by unwholesome provisions—cold and moist atmosphere—sometimes, by a hot and humid climate. This etiological sketch is sufficiently correct. Our author observes that, when the symptoms of inflammation are the preponderating ones, the disease is simply termed enteritis—when the motions do *not* contain blood, it is diarrhœa—but when the dejections are bloody, accompanied by griping and tenesmus, the term dysentery is applied. We consider this to be loose, and somewhat incorrect pathology. Our observations and dissections have led us to conclude that, in proportion as the force of the inflammation was directed upon the peritoneal and muscular coats of the intestines, constipation prevailed—and, on the contrary, when irritation or inflammation was seated in the mucous membrane, diarrhœa or dysentery obtained.

Enteritis may prove fatal in a very few days—it may last a fortnight—and, finally, it may pass into a chronic state, and produce various organic lesions of the bowels. When the chronic phlogosis, for instance, has continued for a certain time in the mucous membrane, a disorganization is always very perceptible after death—generally in the form of a thickening of the membrane, sometimes of several inches in extent.

*Case* 1. A little boy, seven years of age, was taken ill on the 1st of June, 1818, after a sudden mental emotion, and entered the Hopital des Enfans on the 11th of the same month, presenting the following, among other symptoms, viz. face flushed—violent head-ache—mouth dry—tongue yellow, and red in the middle—urgent thirst—frequent cough—pain in the right side of the thorax—abdomen tense and painful—skin dry and hot—pulse quick and sharp—diarrhœa. The remedies consisted of emulsions, a few leeches, and an occasional lavement. The symptoms persisted, with very little alteration, till the 22d of June, when death took place, Delirium had been a prominent symptom during the greater part of the time.

*Dissection.* The membranes of the brain were perfectly healthy—the substance rather firm, but not injected—a very small quantity of serous fluid in the lateral ventricles. The thoracic viscera were in a state of perfect integrity. The mucous membrane of the stomach was red, inflamed, but not disorganized. The mucous membrane of the intestines presented several patches of deep ulceration, and numerous fungous granulations, especially near the cæcum: the other abdominal organs were sound.

In the above case we see an example of violent disorder of the head and chest, without any trace of inflammatory action on dissection. There is, therefore, every reason to believe that the functions of the head and lungs were deranged from that intimate sympathy which exists between the abdominal viscera (especially their mucous surfaces) and the sensorium, heart, respiratory apparatus, &c.

Two cases are next related by M. Tacheron, where there was unequivocal enteritis—one of colonitis—cured by very gentle means—but in each case a cutaneous eruption took place, which evidently assisted the curative measures.

*Case* 2. A female, aged 62 years, had been subject for six years (since the cessation of the catamenia) to an erysipelas, which came out every month, on some part of the body, and lasted four or five days. Latterly, this erysipelas was superseded by a periodical epistaxis—and this last had, also, ceased for some months, without any apparent substitute. On the other hand, she became subject to strong palpitations, violent head-aches, pains in the abdomen, (where she had an umbilical hernia) loss of appetite, inability to lie on her back or left side without sense of suffocation, difficult breathing, small and quick pulse, flushed face, thirst, dryness of skin, general debility. These symptoms continued, or increased, for ten days after the patient's entry into the hospital, and she then died in a paroxysm of suffocation.

*Dissection.* The pleura, on the left side, was adherent to the lungs, and somewhat thickened. The parenchyma of the lungs gorged with blood. The bronchia slightly phlogosed. The heart was enlarged, the right ventricle attenuated in its parietes, the lining surface of which was the color of lees of wine. Liver sound. Stomach striped on its internal surface; several black patches, under which were found ulcerations of the mucous membrane of the small intestines—a large ulceration in the posterior part of the cæcum. The body of the uterus scirrhous—the cervix gorged and red, but still soft, with a tubercle, the size of a nut, imbedded in its substance. On the upper part of the right ovary was a transparent cyst, the size of a pigeon's egg.

The above presents a melancholy picture of what too frequently occurs to females, after the turn of life. Much might be done in these cases, if taken early, by regulated diet, attention to the functions of the digestive viscera, and gentle, but periodical evacuations, till the balance of the constitution became regularly poised.

*Case 3.* Robert Filiol, (in M. Chomel's wards) 32 years of age, had been subject, about the period of puberty, to a dartrous eruption around the umbilicus, which recurred and disappeared, alternately, for several years. On the 6th of April, 1816, being exposed, on a journey, to great vicissitudes of heat, cold, and wet, he felt extreme lassitude, followed by anorexia, pain and tension in the abdomen, with constipation, inclination to vomit, and vomiting of every thing swallowed. The urine was much diminished in quantity. These symptoms went on, without intermission, by day and by night, attended with great emaciation, till the 19th of the same month, when he presented the following phenomena: *viz.* The pain in the abdomen, though not so acute as it had been, was still severe, and was always exasperated by swallowing any thing. The inclination to vomit, and the gazeous eructations continued, as, also, the most complete constipation. The abdomen was very much swelled, and a fluctuation could be felt. The skin was yellowish—the countenance was expressive of great distress—the pulse small, quick, and weak. These symptoms went on, and the patient, in two or three days from this period, died.

*Dissection.* The jejunum was found enormously dilated, and a kind of cylindrical tumour containing an immense volvulus, or rather an invagination, presented itself at the lower extremity of this dilated portion of intestine. The invaginated folds of bowel were glued together by adhesive inflammation. At the commencement of the invagination there was found a small oblong tumour growing out from the internal surface of the gut, in the shape of one's thumb, and this doubtless caused an obstruction to the passage of the fæcal remains along the gut, with dilatation, and the subsequent volvulus.

*Case* 4. Bernard, 33 years of age, had felt, for eighteen months, a dull pain in the right hypochondrium, which often obliged him to incline his body to that side, but which occasioned no particular alteration in his health. In addition to the above, he became suddenly affected with sharp pains about the navel and kidneys, which he compared to a bar through the abdomen. From this time he presented the following symptoms, viz. loss of appetite; eructations after eating, but without nausea or vomiting; dull pain in the epigastric region a little to the right, increased after eating; borborygmi; habitual constipation; great flatulence, forming sometimes a kind of ball in the abdomen, after the dispersion of which there were lancinating pains in the part. He now entered the Hotel Dieu, where he continued two months, with little variation from the above state, except progressive emaciation. He got tired, and took his departure from the Hotel Dieu, and shortly afterwards entered La Charite, where the following phenomena were registered. Two or three hours after meals a pain in the region of the kidneys—unpleasant sense of fulness in the stomach—a prominence in this part —eructations—inclination to vomit, and soon afterwards actual vomiting of greenish matters mixed with the food. These vomitings took place almost every day from one to five or six times, followed by cessation of the sense of fulness in the epigastric region. During this time the dejections were difficultly procured, and composed of hardened matters, at first black, and afterwards yellowish. The emaciation made great progress. From this time, a tumour, very hard to the touch, was abundantly evident in the epigastric region. The symptoms above described continued for several months, till, worn out by his sufferings, he at length was happily released from this cruel state of existence.

*Dissection.* The thoracic organs were sound. In the abdomen was some blackish fluid Nothing particular was observable in the stomach. The duodenum was of a black colour, mixed with red points. Its coats were about half an inch in thickness, and, when cut into, presented a lardaceous appearance. The interior of this organ did not appear so much diseased as the exterior. Its cavity was large, and its folds very prominent. The duodenum, which was itself scirrhous, was projected forwards by a tumour springing from the mesentery, twice the size of a man's fist, of an albuminous nature, and very compact consistence, having contracted adhesions with the spleen and pancreas, but without tendency to suppuration or ulceration. The pylorus was sound, the liver black, and the gall-bladder gorged with green bile.

Cases of this kind are exceedingly puzzling to the practitioner, and often lead him into false prognosis and diagnosis, as we have many times seen. Every dissection, as connected with the history of the case, is therefore very valuable.

*Case* 5. Chevalier, 42 years of age, had been subject for a long time to epistaxis, which became suppressed, and was followed by cerebral congestions, relieved by frequent blood-letting. In the month of September, 1816, he became affected with constant pain in the stomach, bad taste in the mouth, indigestion. In the beginning of January, 1817, the pains in the stomach increased—the abdomen became swelled—the digestion was extremely difficult—flatulence distressing; but no vomiting or constipation. Admitted into the hospital on the 5th of January, he presented the following phenomena, viz. pallid countenance; air of suffering; restlessness; distention of the abdomen; epigastric region shining and tender; diarrhœa; pulse small and frequent. In a few days after his entry into hospital the patient experienced an attack of gastro-intestinal irritation, recurring every second day. At this time a small tumour could be felt in the left hypochondrium. By the end of the month the gastro-intestinal irritation had subsided; but some cough came on. On the 5th of February the symptoms were reproduced, with unusual pain in the epigastrium. There was cough, with oppression on the chest; expectoration; great pain in the epigastrium, with more prominence of the tumour. In the beginning of March there was a temporary amelioration of the gastro-intestinal irritation, cough, and epigastric pain, which continued for three weeks, when great distention of the abdomen and oppression recurred. In the commencement of April all the symptoms attained a great degree of aggravation, accompanied by difficult respiration, cough, and copious expectoration of a purulent kind. On the 10th he died, in a state of great emaciation.

*Dissection.* The chest sounded well on the left, but badly on the right side. In this side of the thorax there was an effusion of three pints of serum, the pleura costalis and pulmonalis being very red, and slightly thickened. The lung of this side hepatized, and condensed to one-third of its ordinary volume. The bronchia were red and filled with mucosities. All the abdominal viscera were confounded in a mass which it was most difficult to unravel. The peritoneum was the seat of innumerable tubercles, many of them in a state of suppuration. The great epiploon was in a state of scirrhous thickening, and adherent to the stomach and liver. All the intestines were glued together, and fixed in their places immoveably.

Such cases as the above are not of very rare occurrence. We have been present at, at least, a dozen of such dissections, and the treatment of some of the cases fell under our own care. These lesions are, in our opinion, the products of inflammation modified by some peculiarity of constitution not yet clearly understood. Dr. Baron would class the above disease, we apprehend, under the head of tuberculous accretions of an hydatid origin.

*Case* 6. Antoine Gaillard, 48 years of age, had been, for some years, subject to stomach complaint. About a year ago he became affected with violent colics, accompanied by bloody stools. These symptoms got worse, and he suffered dreadful pain whenever evacuations took place from the rectum. About six months ago he passed a large quantity of black fetid blood by stool, which seemed to mitigate, for a time, his sufferings. But soon after they returned with such intensity that he could not pass a motion without uttering the most piercing cries. He was received into the hospital on the 9th of February, offering the following symptoms, viz. pale and emaciated countenance—abdomen swelled and tender, especially in the hypogastric region—no appetite—digestion tolerably good—stools liquid and bloody, and passed with great pain, and sensation of a tumour in the rectum—difficulty in making water—fever. These symptoms went on increasing till the 23d of March, when he died.

*Dissection.* The thoracic organs sound. Liver, stomach, and small intestines healthy—large intestine filled with hard fæcal matters—the rectum greatly dilated, and at an inch from the anus a large cancerous ulcer three inches in extent, with hard and elevated edges. The parietes of the gut were scirrhous.

We have passed over a great number of cases where recovery took place; because continental treatment can create little interest on this side of the channel. We have, on the other hand, presented all the more interesting cases where dissection came to the elucidation of the pathology of the disease. We now come to the urinary organs.

Sect. II. *Catarrhus Vesicæ.* This is defined by M. Tacheron, to be inflammation of the lining or mucous membrane of the ureters, bladder, and even the urethra, attacking men rather than women—and old age, rather than the younger periods of life. Among the causes, he enumerates the use of cantharides, acrid diuretics, urethral hæmorrhages, colds, and suppression of the perspiration, retropulsion of cutaneous maladies, violent mental agitations. There are other causes which produce chronic vesical catarrh, viz. a stone in the bladder, or other foreign body—abuse of bougies—enlargement of the prostate gland—anxiety of mind—sedentary habits—gonorrhœas—abuse of venereal indulgences, and any other cause which determines an inordinate irritation to the parts in question.*

The acute vesical catarrh usually continues twenty or thirty days, ending in resolution, or, what is more frequent, in chronic catarrh, subject to irregularities in strength and duration, but generally very obstinate in bending to treatment. Two cases of acute vesical catarrh are related, and both terminated safely. We shall give a short abstract of these.

*Case* 7. Louis Gaille, 30 years of age, of strong constitution, was seized on the 4th of April, 1817, after exposure to cold when heated, with shiverings, pains in the back and hypogastric regions, soon after which he felt acute pain in making water, the urine presenting a flocculent and glairy sediment. He entered the hospital on the 10th, when the following symptoms were noted, viz. head-ache—abdominal pains, with inclination to make water—thirst—constipation—great pain in making water—frequent erections and involuntary emissions of semen—pulse full and frequent—hypogastrium painful on pressure, and tense. Ten leeches to the anus—semicupia—ptisans—aperients—enemata. From the 11th till the 12th very little alleviation of the symptoms. 13th. The abdominal pains less acute; but in the perineum they were very violent. These were relieved by an emollient cataplasm to the perineum, and warm glysters. The symptoms progressively declined, and he was discharged cured on the 27th of April.

*Case* 8. Alexander Crance, 32 years of age, had had blennorrhagia for some years, which was suddenly sup-

* We would just hint to M. Tacheron that he has copied almost verbatim the words of Renauldin in this place, without acknowledgment.—*Rev.*

pressed in April 1818. On the 22d of May he entered the Hotel Dieu, presenting the following symptoms, viz. difficulty in making water—sense of burning heat at the neck of the bladder, after the discharge of urine—tension and swelling of the hypogastrium. The catheter was introduced, and drew off a large quantity of urine, depositing a copious glairy sediment. Diluents, warm baths, &c. for six days. The symptoms gradually declined, and terebinthinate medicines were usefully employed when the inflammation had subsided.

*Case* 9. *Chronic Catarrhus Vesicæ.* Raphael Bouvier, 52 years of age, was affected at the age of 30, with acute pains in the bladder, propagated to the loins, with dysury, mucous, glairy, and gravelly urine. These symptoms, he said, declined in a month under diuretic medicines; but they afterwards recurred, from time to time, with less intensity, and always giving way to remedies. In the month of May, 1811, the symptoms returned with more intensity than ever, and the usual remedies failed. Received at the Clinique the 29th of June, 1811, he presented the following symptoms, viz. frequent desire to make water, with difficulty of expelling it—great quantity of glairy sediment in the urine—constant pain in the bladder. The catheter was introduced—diuretics—emollients—lavemens—warm baths. The latter alone gave any relief. The complaint did not make progress till the middle of July, from which period the strength declined—the appetite failed—the inclination to make water became constant—sleep vanished—pulse became small and frequent—delirium supervened—and death closed the scene on the 1st of August.

*Dissection.* The bladder, when removed from the body, preserved its conical form, and offered great resistance to the pressure of the fingers. Its volume was considerable, and its colour a brownish red. When cut open a large quantity of yellowish purulent looking fluid, of a very disagreeable odour, issued. The parietes of the organ were of an extraordinary thickness and density. The ureters were greatly dilated.

*Case* 10. Gautrin, 52 years of age, had been affected with blennorrhagia for several years, but until November, 1816, he did not experience much inconvenience. At this period the water became ropy and mucous, with pain and difficulty in rendering it—the abdomen was sensible to pressure. A sound was kept pretty constantly in the urethra for a month. Each time it was withdrawn the urine flowed freely, After this period the quantity of glairy mucus increased more than ever, and he entered the Hotel Dieu on the 19th May, 1818, presenting the following symptoms, viz. urine scanty, ropy, and mucous, passed with difficulty—diarrhœa—abdomen tense and painful—features sharp—tongue dry—cough, and pain in the left side—breathing embarrassed—pulse full and strong. Catheterism—mucilaginous drinks—warm bath. 20th. Abdomen more painful—diarrhœa increased. Twenty leeches to the hypogastrium. No amelioration of the symptoms—the respiration more difficult. 25th. All the inflammatory symptoms much exasperated. *Four leeches to the hypogastrium*—diluents—lavemens, &c. 26th. Constant groaning. 27th. Twenty leeches to the chest—a blister. Died in the evening.

*Dissection.* Several ounces of pus in the peritoneal cavity, and several false membranes adherent to the small intestines—the internal surface of the bladder was of a dark colour, and in its parietes, at the back part, there was a large purulent cavity capable of holding a pullet's egg, situated between the muscular and mucous tunics, communicating with the general cavity of the bladder by several openings, through one of which the point of the sound had passed. The commencement of this false passage was discovered on the right side of the prostate gland, which was of double the natural size. The kidneys were tuberculated.

*Case* 10. Louis Devaux, 74 years of age, experienced in June 1817, slight pains in the loins, lower extremities, and hypogastric region, with frequent desire to make water, and pain about the neck of the bladder. Towards the middle of October of the same year, these symptoms increased, and he began to lose his appetite. On the 30th of October he was received into the Hospice, presenting the following symptoms, viz. fixed pains in the hypogastric region—tongue red at the edges and coated in the middle—bad taste in the mouth—nausea—hiccup—abdomen slightly distended—stools scanty—frequent desire to make water—urine yellowish, and containing much whitish mucus—pulse feeble, and quick. *Emollient drinks.* Slight amelioration of the symptoms. *9th November.* A colliquative diarrhœa—intense thirst—great prostration of strength—tendency to faintness. 20th. Vomits his drink—great heat of skin—pulse very quick—died on the 26th of November.

*Dissection.* Thoracic viscera perfectly healthy—some points of phlogosis in the small intestines—in the colon some actual ulcerations, with high edges—the kidneys, especially the left, of an extraordinary size, with here and there purulent depôts—the pelves of the kidneys were greatly distended with a quantity of sanguinolent and fetid sanies—the bladder contained a similar liquid—its coats four times their natural thickness.*

We have now presented the reader with all the pathological facts brought forward in this volume, as ascertained by dissection. Our author's therapeutical reflexions at the end are few. He recommends the warm bath, mucilaginous drinks, and diluent regimen from the beginning. When the inflammatory symptoms run high, then general and local bleeding is to be employed. Where the canal of the urethra becomes obstructed (which is very often the case) with thickened mucus, the catheter or bougie should be occasionally introduced, to prevent the bladder being irritated by distention and the presence of acrid urine. When the catarrhus vesicæ is dependent on a calculus lodged in the bladder, it is almost needless to observe, that no cure can be expected till the cause is removed by an operation. In people beyond the age of 40—indeed in all people, the bladder should be sounded, in order to ascertain if a calculus be present. We recently saw a melancholy instance of the neglect of this, in the case of a gentleman who was three years under one of the first surgeons in this metropolis, for the treatment of catarrhus vesicæ. During all that time he never sounded his patient, but treated the disease as simply chronic inflammation of the mucous membrane of the bladder. The surgeon-apothecary in the country, where the gentleman resided, long suspected the presence of stone, but would not be permitted to examine, till after three years and more of great torture, when a very large calculus was discovered as the cause of the disease. The gentleman, then, came to town, and put himself under another surgeon, who confirmed the statement of the surgeon-apothecary; but the bladder was in such a diseased state, and there were such strong reasons to fear that actual ulceration existed, that an operation durst not be ventured on! Thus the time was lost for a successful operation by the overweening confidence of the London surgeon in his own diagnostic powers! Let this be a warning not to neglect the examination of the bladder in catarrhus vesicæ, whether the common symptoms of stone be present or not.†

When it is ascertained that a stone is not the cause of the catarrhus vesicæ, then it is of great importance to investigate

---

* Our author has not noticed an appearance in the mucous membrane of the bladder, which we have twice seen—namely, a development of small glands in this tunic, which, when squeezed, pour out a quantity of glairy mucus, and from these glands scattered all over the interior of the bladder, the mucus deposited by the urine undoubtedly came.—*Rev.*

† The patient was operated on while this sheet was passing the press, and some hopes are entertained that the result may still be favourable.

what has been the original cause of the inflammation settling upon that part; for much of the success of the treatment depends on this investigation. Thus, if the patient had experienced a retrocession of some cutaneous eruption, it will be proper to act on the skin, so as to re-establish the irritation on the surface. There is great reason to believe that, in many instances, the vesical irritation is of a gouty or even rheumatic character, in which cases it is evident that the treatment should be greatly modified by a knowledge of the cause.

Catarrhus vesicæ, especially in old people, is very difficult of cure, since there appears to be a natural tendency to disease of the urinary organs in almost all people who have arrived at an advanced period of life. This was an observation of Hippocrates himself—"*Renum et vesicæ dolores difficulter sanantur in senibus.*" Although the mucous and glairy discharge tends greatly to produce debility, while the pain, irritation, and consequent want of sleep help to reduce the patient's strength; yet, when the disease does not depend on stone in the bladder, and is not accompanied by fever or much pain, it may go on for many years, and scarcely abridge the natural range of the patient's life. It is, however, at all times desirable to remove the disease, if possible. For this purpose the diet should consist almost entirely of the farinaceæ, and light animal food—especially the former. Every thing that has a tendency to act on the kidneys should be avoided, as horseradish, mustard, onions, &c. The water drank should be distilled water, as much less irritating to the bladder; and the urine should be frequently tested to ascertain whether it inclines to acidity or alcalescency. In the former case soda should be taken every six or eight hours—in the latter, the mineral or vegetable acids. Soda will, in general, be very useful, especially where there is a stone in the bladder, as it renders the discharge of water infinitely more free and painless. The decoction of uva ursi, (one ounce to the pint of distilled water,) with from half an ounce to an ounce of the tincture of henbane, a wine glassful twice or thrice a day, will be found a very useful remedy. It is of great importance in this disease to regulate the functions of the bowels, and to avoid strong purgatives. The patient should get into the habit of emptying the lower bowels by means of injections, and only use the mildest aperients when necessary. The hip-bath ought not to be neglected. Injections of tepid water, or of water in which opium is dissolved, have often a very beneficial tendency, as the urine frequently acquires a very acrid character, and irritates the already too sensible coats of the bladder. It is doubtful whether any thing more is to be expected from injections than soothing the bladder, and diluting the urine. Some medical men, however, have employed the liq. plumbi acet. dilut. with the view of repressing the discharge of mucus, and it has been said with benefit. Chopart, for instance, relates the case of an old man, who was fast wearing down with an excessive discharge of glairy mucus, and who was greatly benefited by injections of the abovementioned liquid. But, viewing the discharge as a mere effect, and probably a salutary one of the local inflammation, we confess we should not place much confidence in repressing the effect without removing the cause.

### Inflammation of the Serous Membranes.

These membranes, from the similarity of their texture and functions, have a considerable analogy in the nature of their diseases, as well as in the causes and treatment of those diseases. And, although it very seldom happens that the seat of inflammation is long confined to these structures alone, without being extended to the parenchymatous textures which they cover; yet it is undoubted that in the incipient stages of the phlogosis, and sometimes throughout the whole course of it, only the serous covering of an organ or part will be the seat of the disease. The attempt, therefore, to discriminate the symptoms of membranous from parenchymatous inflammations should never be branded with the term "*useless refinement*," as has sometimes been done. It is as proper to ascertain the distinctions in the living as in the dead body—and where the disease is not complicated, this may generally be done. In most cases, however, or in the advanced stages of most cases, the subjacent textures become affected, and these react sympathetically on the functions of various other organs, thus producing obscurity and confusion in the diagnosis.

We must pass over the first two sections of this part of the work, on arachnitis, and on hydrocephalus acutus, in consequence of having treated so fully of these topics in several preceding numbers of the Journal. We must dwell a little on the following subject.

Sect. III. *Pleuritis.* This disease, described ever since the days of Hippocrates, has given rise to many controversies respecting its separate existence. It is well known that Cullen considered it as always complicated with pneumonia, or, at all events, that it could not be distinguished in the living body. But later physicians, and especially Laennec, have been more precise, and have proved, not only the separate existence of pleuritic inflammation, but shewn that it is, in general, accompanied by its appropriate symptoms. In a great majority of instances, however, it is complicated with inflammation of the subjacent structure. Like most other phlegmasiæ it is either acute or chronic. The former is much more easily distinguished than the latter; and yet the symptoms, even of the acute form, are not so unequivocal as might be expected. Thus the pain in the side, the impeded breathing, the fever, the dry cough, are not always pathognomonic of pleuritis; since, on the one hand, they may all exist without the pleura being inflamed;—and, on the other, the pleura may be in a state of phlogosis, without these symptoms being present. Thus, when the inflammation comes on slowly, it does not shew the external phenomena presented during a sudden accession of the disease. Percussion of the chest will bring forth a dull sound, it is true—but then it is only in an advanced stage of the inflammation, and when some degree of effusion has taken place. Even here we cannot tell whether the dull sound proceeds from pleuritic effusion or parenchymatous induration. The pain in the side is equivocal—for we may have pleurodyne independent of inflammation. The hardness of the pulse was considered by Baglivi, who studied, with great care, the diseases of the lungs, as the least equivocal of all other symptoms of pleuritis. "*Pulsus durities est signum fere infallibile omnium pleuritidum. Si duritiem in pulsu deprehenderis, quamvis reliqua signa non adsint, pro certo habeas patientem laborare pleuritide.*" And yet Baglivi carried his diagnostic too far, for the veriest tyro knows that, "all other signs absent," the hardness of the pulse is no more an indication of pleuritis than of hepatitis, or any other inflammation of an interior organ. After all, it is but rarely that we can be deceived in acute pleuritis; but in chronic inflammations of that membrane the greatest difficulty obtains in the diagnosis. In fact, chronic pleuritis, with its accompanying effusions and false membranes, will go on in what may be termed a latent course for months or even years, without any very prominent symptoms. We shall quote a few cases from this section of M. Tacheron's work.

*Case* 11. Lecofre, 70 years of age, received a kick from a horse, which fractured his lower jaw. He was immediately taken to the Hotel Dieu, and there treated for the accident. But the patient was very irregular in his habits and regimen. Sixteen days after his reception in the hospital, he was observed sitting upright in bed, and appeared to be menaced with suffocation if he attempted to lie down. He passed the night in the sitting posture, complaining of slight pain in the right side of the chest, but had no cough. The pulse was small and quick. He died the next day.

*Dissection.* Nearly two pints of yellowish fluid in the right cavity of the chest—the lung of that side compressed, but perfectly sound —the pleura coated with a layer of yellow albuminous matter, a line in thickness, and so organized as to be torn off in flakes. There was no other lesion in any part of the body.

It is quite evident that this albuminous layer and the serous effusion were of old date, although nothing of the kind was suspected. The following case is still more remarkable.

*Case* 12. François Fournier, having enjoyed perfect health previously, was seized on the 12th Vendemaire, after a debauch, with cold chills and difficulty of breathing. On the 14th he entered the Clinique, presenting the following symptoms, viz. face flushed—no headache—eyes injected and watery—respiration difficult—acute pain in the right side on inspiration—frequent cough—sanguinolent expectoration—abdomen tense, especially about the right hypochondrium and epigastrium, where there was tenderness on pressure—pulse small and concentrated—skin a little hot—two contusions, one on the hip, another on the shoulder, received during the debauch abovementioned.—*Ptisans.* Slight amelioration during the first three or four days. 19th. A severe sore throat. *Gargles.* From this time there was great prostration of strength—decubitus dorsalis—strong action of the heart, with irregularity—pulse strong and intermitting. From the 19th to the 23d, there was a slight accession of delirium towards mid-day. He died on the 23d, the expectoration being mixed with blood. There was also blood in the urine.

*Dissection.* The right cavity of the chest contained upwards of three pints of a reddish yellow fluid—the lung of that side enveloped in a very thick false membrane of a yellow colour—the lung itself compressed into a small space between two pouches, filled with pus, one pouch adhering to the mediastinum, the other to the costal pleura—left lung sound—heart natural—liver rather enlarged—all the rest sound.

We have no doubt that, in this instance, the whole of the abovementioned ravages were the consequences of a fortnight's unchecked inflammation.

About fourteen years ago, we had opportunities of seeing, on an extensive scale, the effects of unbridled inflammation among Junot's troops returning from Portugal to France, in crowded transports, and in cold, wet, and stormy weather. They were severely affected with what was then termed *Pneumonia Typhodes,* and no bleeding was thought of. It was a fever with great determination to the lungs, occasioned by the inclemency of the skies, and the bad accommodations in the transports. We saw numerous cases where the ravages, in ten days, were quite equal to those described by Tacheron. The lessons there taught, were of a melancholy but very instructive nature.—The following appears to have been a case of this kind.

*Case* 13. Charles B. 51 years of age, was seized on the 16th of September, 1809, with a cold chill followed by heat, afterwards by headache, and fixed pain in the lower part of the left side of the chest, increased by inspiration. These symptoms continued till the 26th September, when he was received into the Clinique, presenting the following symptoms, viz. suborbital cephalalgia—sleeplessness—tongue dry and black—abdominal region tender—intense thirst—diarrhœa—respiration very much confined—cough severe—great fixed pain in the left side—pulse small, and not much accelerated. *A dozen of leeches to the pained side—infusion of bark, &c.* The symptoms went on increasing, and he died on the 2d November.

*Dissection.* No sound could be elicited from any part of the chest—considerable effusion under the arachnoid in the head—the right lung strongly adherent to the ribs of that side, and its lower half converted into a putrid and disorganized mass—the left lung adherent on all sides, but not affected in its parenchymatous structure—the heart greatly enlarged.

Such examples as the above are fortunately not very frequently seen on this side of the channel, where more active treatment is pursued than on the continent; but they afford useful beacons to warn us of the danger we run in neglecting thoracic inflammation.

Our author next brings forward a number of cases where the pulmonic inflammation was complicated with a similar state of the mucous membrane of the intestines—an occurrence by no means rare.

Section IV. *Hydrothorax.* This disease our author considers as the inevitable consequence of inflammation of the pleura (suite inevitable de l'etat inflammatoire des plevres.) It is generally complicated with, or rather caused by obstinate or neglected catarrhal affections—repeated attacks of convulsive asthma—organic diseases of the heart or lungs. The prognosis may generally be unfavourable—and the event sudden. The following case is interesting, though we think it would be more properly entitled traumatic than idiopathic hydrothorax.

*Case* 14. Jean Jack Lefre, 33 years of age, by trade a blacksmith, received an injury on the right side of the chest, in the month of April, 1818, by a blow from a bar of iron. The pain was great, and there was ecchymosis of the integuments. From this period he felt constant pain in that side, augmented by exercise, with cough in the evenings, sense of weight in the chest, tightness in breathing, general malaise;—but no loss of appetite or sleep. Two months after the accident, he vomited up about a pint of blood, followed by considerable abatement of the symptoms abovementioned. He therefore took to his work, but in a day or two the dyspnœa became more troublesome than ever, and flexion of the trunk almost impossible, with great sense of weight and fulness in the thorax. All these symptoms went on gradually increasing for nineteen months, till the 1st of October, 1819. On this day, the patient felt a remarkable sensation, while making some exertion, attended with a strange noise of something bursting, and with instant sense of suffocation, anxiety, and cold sweats. From this time he could feel a gurgling noise in his chest at each motion of the body, and the symptoms above described continued with great violence. He was received into the Hotel Dieu, on the 18th November, 1819, twenty one months after the commencement of the malady; at which time the symptoms were so unequivocal as to leave no doubt of the nature of the case. No sound could be elicited by percussion from that side of the chest, nor was there any evidence of the respiratory function there. It was just the reverse on the left side of the thorax.

On the 27th December, the operation for empyema was performed by M. Dupuytren, at the request of M. Recamier. A hydrocele trochar was plunged between the fifth and sixth ribs (counting from below) at equal distances from the sternum and spine. Three pints of fluid escaped, at first of a clear serous nature, but towards the end of a turbid and greenish cast. Next day the patient took some hot wine, which brought on a fever of some days duration. On the 29th, the diseased side was more sonorous on percussion than the other. On the 3d of January 1820, the right side of the chest seemed much bulged out—a gurgling noise distinctly heard when the trunk was moved—that side yet more sonorous than the left—no respiration heard there—and but faintly in the other side—the beating of the heart very quick and rather strong—palpitation on any movement of the body. On the 6th of January, the breathing was become very difficult—no sleep—orthopnœa—great debility—extreme anxiety. On the 10th the patient died suffocated.

*Dissection.* On opening the right cavity of the chest, much fetid gas escaped, followed by a quantity of whitish pus, to the amount of three or four pints. The whole internal surface of the pleura was covered by an albuminous exudation. The lung of that side was shrivelled up into a very small space, but still a little crepitous—no communication between the cavity of the pleura and the bronchia—the mucous membrane of the aerial passages presented traces of inflammation. Nothing remarkable in the other side of the chest. All the other organs sound.

Our author thinks there is reason to believe that the fluid was originally contained in a cyst, which cyst burst at the time the patient felt the unusual noise and sense of suffocation detailed above.

In the following case, which is sufficiently interesting, we attribute the sudden death to disease of the heart, and effusion into the pericardium, rather than to the hydrothorax of the pleura.

*Case* 15. A young woman, 26 years of age, who had been a recluse in the Saint Lazar for several years, was admitted into the Infirmary on the 18th of May, complaining of pains in her limbs, fever, and severe cough. On the 19th an emetic was administered, and, with the exception of the cough, the patient felt herself better. This symptom continued with violence, but without any oppression of the chest, or pain in the side. There was some evening fever. This state continued till the 24th, when flushing of the face, dryness of the tongue and skin, bad taste in the mouth, nausea, &c. returned, and another emetic was administered with the same reults as on the former occasion, excepting the cough, which still continued. On the 26th the patient could lie on either side, but with preference for the right—the evening fever returns—the stools and urine scanty. 29th. Pains in the limbs—and at intervals a pulsation in the epigastric region—some blood in the expectoration—bad sound, on percussion, from the inferior part of the sternum. Between the 30th of May and 1st of June there were bilious vomitings, with tendency to faintings—the epigastrium very tender to the touch. On the 3d of June, in the morning, the patient appeared strangely affected, and in a manner which she could not describe. She wept; and yet she seemed better in all respects than for a considerable time previously. The complexion was natural—the features exhibited no traits of suffering—the pulse was calm, full, and regular—the skin cool and moist—the tongue clean—the breathing easy—no cough, nor pain in the chest. In the evening she got up, and after walking about a little and talking jocosely with some of her companions, she fell down dead, while returning to her bed.

*Dissection.* The right side of the thorax presented an effusion, circumscribed and encysted, as it were, by a membrane formed of the pulmonary and costal pleuræ, and containing about a pint and a half of yellowish transparent serum. The parietes of this cyst were as red as blood, and highly inflamed. The pleura covering the diaphragm, throughout its whole extent, was very much thickened—in some places to that of four lines. The lung of this side was flattened and hard—in some few places a little crepitous. The left lung was sound, and no effusion on that side. The heart was greatly enlarged, and the arch of the aorta extremely dilated. The pericardium contained some ounces of water. The small intestines were slightly phlogosed—the mucous membrane of the stomach was red, and its vessels strongly injected.

It is observed by M. Tacheron, and with reason, that the serous collection here was the result of chronic inflammation of the pleura; but the effusion was so slow that the neighbouring lung accustomed itself to the gradual encroachment; hence the obscurity of the symptoms. We conceive that the sudden death was owing to the effusion, though trifling, into the cavity of the pericardium. It is astonishing what a small effusion into that cavity will do, when there is any organic disease of the heart, or water in any of the other thoracic cavities embarrassing the function of the lungs.

The following is a well marked case of tubercular disease, pervading many parts of the body at the same time.

*Case* 16. Antoinette Rose, 16 years of age, had enjoyed good health till the age of 13, when she was seized, in the month of May, 1806, with a gastro-intestinal phlegmasia of an intermittent character, accompanied by some cough, which continued till her entrance into the hospital. She had some difficulty of breathing—pain and hardness in the left hypochondrium. These symptoms were generally relieved every day when the fever came on, and returned in the apyrectic intervals. She sometimes coughed up a little blood with the expectoration, and twice had considerable nasal hæmorrhages, followed by much alleviation of the symptoms. On the 5th of December, 1806, she entered the hospital, presenting the following symptoms:—face pale and emaciated—constant thirst—cough—pain to the left of the epigastrium—abdomen tender on pressure, and a tumour to be felt in the left hypochondrium—liver somewhat enlarged and indurated—diarrhœa for some days past—pulse feeble and quick. This state continued from the 5th till the 31st December;—the patient often complaining of head ache—great heat in the palms of the hands—puriform expectoration—heat of skin—hard pulse. On the 8th of December twelve leeches to the pudendum—diluents, &c. From the 1st till the 15th of January the cough and diarrhœa somewhat mitigated. From the 15th till the 31st an eruption on the left arm and hand—considerable fever. From the 1st till the 18th of February the abdominal pains more distressing, and the pyrexial symptoms increased—diarrhœa constant—legs œdematous. From the 18th till the 25th her state was very alarming—the breathing very short—voice plaintive—pulse small—skin arid and hot. Death put a period to her sufferings on the 28th of the same month.

*Dissection.* The thorax, when stricken, resounded but badly on the left side—fluctuation in the abdomen. The right lung was sound and crepitous—a serous effusion on the left side, with here and there adhesions between the costal and pulmonary pleura—the pleura itself studded with granulations and small tubercles—the lung itself but little crepitous, yet with no particular disorganization—the heart remarkably small—effusion in the abdomen—the whole peritoneal covering of the intestines studded with black granulations and various sized tubercles, the smallest of which were the magnitude of a pea—the mesenteric glands enlarged—all the circumvolutions of the intestines adherent to each other—liver greatly enlarged, tuberculated, and very hard—the gall-bladder contained but a small quantity of limpid fluid—the fallopian tubes enlarged and tuberculated.

We have not space to bring forward any more dissections under the head of hydrothorax. We may be pretty well satisfied, that these effusions into the thoracic cavities are the consequences of acute or chronic inflammation, or of some obstruction to the free circulation of the blood. Although these effusions are serious evils in themselves, they are far less so than the causes which produce them—and it is to the removal of these that we are to look for any thing like a permanent cure. Unfortunately, it too often happens that we have little power over any thing but this *effect* of the disease, the effusion. We can frequently evacuate this, by acting on the absorbent and secerning vessels, and thus give a temporary relief to the sufferings of the patient, when the organic disease, that is the cause of all, is beyond our reach.

A series of important pathological specimens still remain for examination; but as they belong to distinct classes, they will form one or more moderately sized articles in our succeeding numbers, without producing any inconvenience in their separation from the present paper. They are principally on diseases of the pericardium, heart, peritoneum, brain, and nervous system. They will appear in due time. Meanwhile, we invite our junior brethren to cultivate pathological anatomy with increased energy and ardour, as the only safe basis of practical medicine.

